# DNAzyme-Functionalized R-Phycoerythrin as a Cost-Effective and Environment-Friendly Fluorescent Biosensor for Aqueous Pb^2+^ Detection

**DOI:** 10.3390/s19122732

**Published:** 2019-06-18

**Authors:** Jikui Wu, Yunfei Lu, Ningna Ren, Min Jia, Ruinan Wang, Junling Zhang

**Affiliations:** 1 College of Food Science and Technology, Ministry of Agriculture, National R&D Branch Center for Freshwater Aquatic Products Processing Technology (Shanghai), Laboratory of Quality and Safety Risk Assessment for Aquatic Product on Storage and Preservation (Shanghai), Shanghai Ocean University, Shanghai 201306, China; yifland0929@163.com (Y.L.); Ningna_ren@163.com (N.R.); m170200397@st.shou.edu.cn (M.J.); m170200399@st.shou.edu.cn (R.W.); 2 Laboratory of Freshwater Aquatic Genetic Resources, Ministry of Agriculture, Shanghai Engineering Research Center of Aquaculture, National Demonstration Center for Experimental Fisheries Science Education, Shanghai Ocean University, Shanghai 201306, China

**Keywords:** DNAzyme, R-phycoerythrin, sensors, Pb^2+^, fluorescence resonance energy transfer

## Abstract

The sensitive detection of Pb^2+^ is of significant importance for food safety, environmental monitoring, and human health care. To this end, a novel fluorescent biosensor, DNAzyme-functionalized R-phycoerythrin (DNAzyme-R-PE), was presented for Pb^2+^ analysis. The biosensor was prepared via the immobilization of Iowa Black^®^ FQ-modified DNAzyme–substrate complex onto the surface of SPDP-functionalized R-PE. The biosensor produced a minimal fluorescence signal in the absence of Pb^2+^. However, Pb^2+^ recognition can induce the cleavage of substrate, resulting in a fluorescence restoration of R-PE. The fluorescence changes were used to measure sensitively Pb^2+^ and the limit of detection was 0.16 nM with a linear range from 0.5–75 nM. Furthermore, the proposed biosensor showed excellent selectivity towards Pb^2+^ even in the presence of other metal ions interferences and was demonstrated to successfully determine Pb^2+^ in spiked lake water samples.

## 1. Introduction

Lead ion (Pb^2+^) is a highly toxic heavy metal that has significant adverse effects on food, the environment and human health [1,2,3]. It is not biodegradable and easily accumulated in a living organism. Therefore, developing the analytical methods for accurate monitoring of Pb^2+^ is important for food safety, environmental monitoring, and human health care. The traditional Pb^2+^ detection methods include atomic absorption spectrometry (AAS) [4], X-ray fluorescence spectrometry [5], electrochemical analysis [6,7], and inductively coupled plasma mass spectrometry (ICP-MS) [8]. However, most of these methods often require time-consuming sample pretreatment, expensive instruments, and well-trained personnel. As a consequence, there is an urgent need to develop simple, low-cost and sensitive assays to determine Pb^2+^ in environmental and biological samples.

DNAzymes are catalytic single-stranded DNA sequences with high specificity towards metal ions [9,10,11]. Due to low cost, excellent selectivity and easy storage, DNAzymes have attracted significant attention as a promising recognition element for sensing metal ions [12,13,14,15]. In the past decade, researchers have exploited 8-17 DNAzyme or GR-5 DNAzyme to design the labeled fluorescence sensors for Pb^2+^ detection [16,17]. Conventional DNAzyme-based labeled fluorescent biosensors are usually constructed by labeling a fluorophore at the 5’ end of the substrate strand and an organic quencher at the 3’ end of the enzyme strand, respectively [18,19]. However, these biosensors are limited in practical applications because of their high background fluorescence. To improve lead-detection sensitivity, organic quenchers were replaced with nanoquenchers such as carbon nanotubes [20], gold nanoparticles [21,22], and graphene [23,24] to decrease the fluorescence background. Furthermore, Fan et al. utilized the superior optical properties of quantum dot to enhance the detection sensitivity for Pb^2+^ assays [25]. Nevertheless, the preparation of nanomaterials is complicated and time-consuming, and some of them are even toxic. Thus, it was worthwhile to explore a novel fluorophore with desirable properties in terms of cost, sensitivity, and environmental protection.

R-phycoerythrin (R-PE) is a stable and low-cost fluorescent protein isolated from red algae [26,27]. It exhibits extremely bright red-orange fluorescence with extremely high absorbance coefficients and high quantum yields. Long-wavelength fluorescence emission (575 nm) of R-PE minimizes interferences from biological molecules in the sample matrix. Therefore, in fluorescence-based detection, the sensitivity of R-PE is usually 5 to 10 times greater than that of traditional organic dyes. Furthermore, R-PE is non-toxic and environment-friendly. These unique properties make R-PE an ideal fluorescent reporter for designing DNAzyme-based fluorescent biosensors. Herein, we employed R-PE as a fluorescent reporter to construct a cost-effective, environment-friendly, and highly sensitive biosensor for Pb^2+^ detection by immobilizing quencher-labeled GR-5 DNAzyme-substrate complex on R-PE (DNAzyme-R-PE).

## 2. Materials and Methods

### 2.1. Materials

R-phycoerythrin (R-PE) was purchased from Langya Biotechnology Co., Ltd. (Shanghai). Dimethyl sulfoxide (DMSO) (≥ 99.9%), tris-(2-carboxyethyl)-phosphine hydrochloride (TCEP) (≥ 98%), *N*-succinimidyl 3-(2-pyridyldithio)-propionate (SPDP) (≥ 95%), HEPES (≥ 99.5%), Lead(II) acetate trihydrate (99.999%), Amicon 30-kDa and 3-kDa cutoff filter (0.5 mL) were obtained from Sigma. 

GR-5 Pb-Enz (5′-ACA GAC ATC ATC TCT GAA GTA GCG CCG CCG TAT AGT GAG-3′-Iowa Black^®^ FQ) and Pb-Sub (SH-(CH_2_)_6_-5′-CTC ACT AT rA GGA AGA GAT GAT GTC TGT -3′- Iowa Black^®^ FQ) were synthesized by IDT (Coralville, IA) and purified by HPLC.

### 2.2. Instrumentations

The concentration of R-PE and DNAzyme-substrate complex-functionalized R-phycoerythrin (DNAzyme-R-PE) was determined by a NanoDrop 2000c spectrophotometer (Thermo Scientific, Waltham, MA, USA). The fluorescence spectrum of R-PE was performed on an FS5 spectrometer (Edinburgh Instruments, Livingston, UK) at an excitation wavelength of 535 nm. Transmission electron microscopy (JEOL-JEM 2010 microscope, JEOL, Ltd., Tokyp, Japan) was used to characterize the size of R-PE. A negative stain of the sample was performed to increase the contrast for imaging. The hydrodynamic sizes of R-PE and DNAzyme-R-PE were measured with a Malvern Nano ZS90.

### 2.3. Preparation of DNAzyme-R-PE Sensors 

R-PE was purified using a 0.5-mL Amicon filter (30-kDa cutoff) and resuspended in HEPES (50 mM, pH 7.5) buffer. The resultant R-PE was activated using SPDP as described in the literature [28]. In brief, 10 μL of 100 μM fresh SPDP stock solutions (in DMSO) was mixed with 25 μL R-PE (4 μM) solution. Next, the addition of 1 μL NaHCO_3_ (1 M) adjusted the pH of the mixture solution to 8.5. The mixture solution was wrapped with aluminum foil and incubated for 2 h at 25 °C, and then centrifugal filtration was used to remove the excess SPDP. The SPDP-modified R-PE was further treated with 0.1 mM TCEP for 30 min at 25 °C. The activated R-PE was further purified by removing the excess TCEP. The concentration of the activated R-PE was determined by a NanoDrop 2000c spectrophotometer (Thermo Scientific, Waltham, MA, USA). 

For the preparation of Pb^2+^-specific DNAzyme-substrate complex, Pb-Sub and Pb-Enz (1:1.2) were mixed in HEPES buffer (50 mM, pH 7.5, 50 mM NaCl). The excess of Pb-Enz was used to ensure the complete hybridization of the Pb-Sub strand by the Pb-Enz. The mixture was hybridized in a 65 °C water bath for 5 min and gradually cooled down at room temperature for 1 h. The resultant Pb^2+^-specific DNAzyme-substrate complex was conjugated to the aforementioned activated R-PE [29].

The activated R-PE reacted with thiol-modified Pb^2+^-specific DNAzyme-substrate complex in HEPES (50 mM, pH 7.5, 50 mM NaCl) buffer at a molar ratio of 1:15 for 6 h at 4 °C in the dark. The excess DNAzyme-substrate complexes were removed by centrifugation with an Amicon 0.5-mL 3-kDa-cutoff filter. The DNAzyme-R-PE was washed twice with HEPES buffer (50 mM, pH 7.5) containing 1 M NaCl, and HEPES buffer (50 mM, pH 7.5), respectively. The DNAzyme-R-PE was resuspended in HEPES buffer (50 mM, 50 mM NaCl, pH 7.5) at a final concentration of 4 μM and stored at 4 °C in the dark until its use.

### 2.4. Gel Electrophoresis Characterization of DNAzyme-R-PE

8% native PAGE was used to characterize the formation of DNAzyme-R-PE, employing R-PE as a control. 10 μL 100 nM DNAzyme-R-PE (or R-PE) was loaded on the 0.75 cm polyacrylamide gel. Electrophoresis was performed at 150 V for ~ 3–4 h at room temperature in 1×TBE buffer. The gel was imaged with the Bio-rad gel imaging system

### 2.5. Fluorescent Assay of Pb^2+^

2 μL of the 500 nM DNAzyme-R-PE was mixed with 1 μL Pb^2+^ with different concentrations. The mixture was then diluted to 100 μL by HEPES (50 mM, 50 mM NaCl, pH 7.5) buffer. After a reaction for 20 min at 25 °C, the mixture was quenched by 3 μL quencher (50 mM EDTA and 8 M urea). The fluorescence intensity of the mixture at 575 nm was then recorded. 

### 2.6. Determination of Pb^2+^ in Real Water Samples

Real water samples were collected from Mirror Lakes in Shanghai Ocean University Campus (Shanghai, China). The samples were centrifuged at 10,000 g for 15 min and filtered with a 0.22 μm filtered membrane. The resultant water samples were spiked with 5, 15, 45 nM Pb^2+^, respectively. DNAzyme-R-PE (10 nM) was incubated with the Pb^2+^-spiked lake water in 100 μL HEPES (50 mM, 50 mM NaCl, pH 7.5,) buffer for 20 min. The reaction was quenched and fluorescence of the water samples were determined at 575 nm 

## 3. Results and Discussion

### 3.1. The Sensing Principle of DNAzyme-Substrate Complex-Functionalized R-Phycoerythrin (DNAzyme-R-PE)

**Scheme 1:**
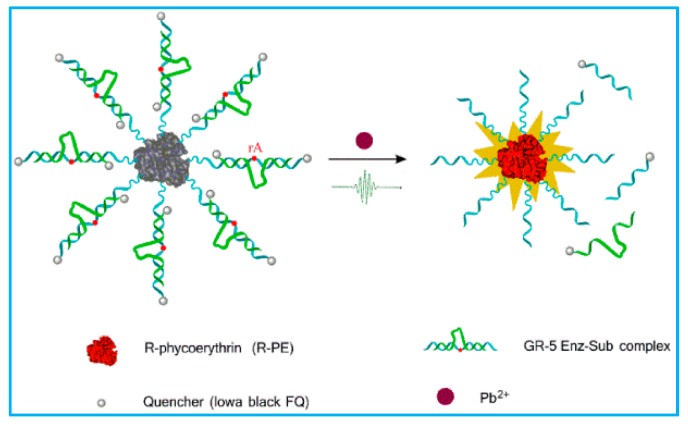
The sensing strategy of DNAzyme-R-PE for Pb^2+^ detection.

As illustrated in Scheme 1, DNAzyme-R-PE consisted of R-phycoerythrin (R-PE), GR-5 enzyme, and its corresponding substrate. R-PE was activated by N-succinimidyl 3-(2-pyridyldithio) propionate (SPDP). The substrate was labeled with a thiol group at the 5′ end and an Iowa Black**^®^** FQ molecule at the 3′ end, respectively. The 3′ end of the enzyme was also modified with Iowa Black**^®^** FQ molecule. DNAzyme-R-PE was prepared by the immobilization of the annealing enzyme-substrate complex onto the surface of R-PE through the s-s bond. Immobilization brings the Iowa Black**^®^** FQ molecules and R-PE into close proximity. Hence, the fluorescence of R-PE was efficiently quenched by the Iowa Black**^®^** FQ molecules. The recognition of Pb^2+^ facilitated the cleavage of the substrate by the GR-5 enzyme. The cleaved substrate and GR-5 enzyme released from R-PE. Thus, the fluorescence of R-PE was restored, signaling the concentrations of Pb^2+^.

**Figure 1:**
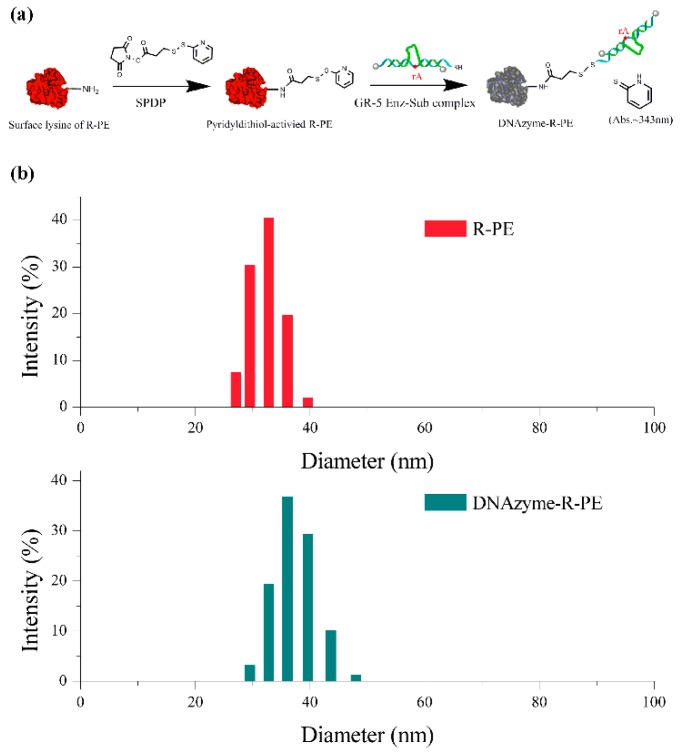
(**a**) The reaction process of the preparation of DNAzyme-R-PE. (**b**) Dynamic light scattering (DLS) spectra of R-PE and DNAzyme-R-PE.

R-PE is a stable and water-soluble globular protein with an average size of 32.6 ± 2 nm (Figure S1). We exploited SPDP, a bifunctional crosslinker, to activate R-PE [28]. SPDP-activated R-PE is then conjugated to the thiol-modified enzyme-substrate complex via a disulfide bond [29], thereby constructing the DNAzyme-R-PE sensor (Figure 1a). We employed dynamic laser scattering (DLS) to determine the hydrodynamic size of R-PE and DNAzyme-R-PE, respectively (Figure 1b). Bare R-PE has a diameter of ~33 nm, which was consistent with the TEM observation. After the modification with thiol-modified enzyme-substrate complex, DNAzyme-R-PE is ~38 nm in diameter. An obvious increase in the size of R-PE was observed, suggesting the successful conjugation of the enzyme-substrate complex onto R-PE [30]. Meanwhile, the native PAGE (8%) experiment indicated the coupling of the enzyme-substrate complex to R-PE, which led to a clear difference in the band shift between R-PE and DNAzyme-R-PE (Figure S2) [31]. This further confirmed that the enzyme-substrate complex was successfully immobilized on R-PE.

**Figure 2:**
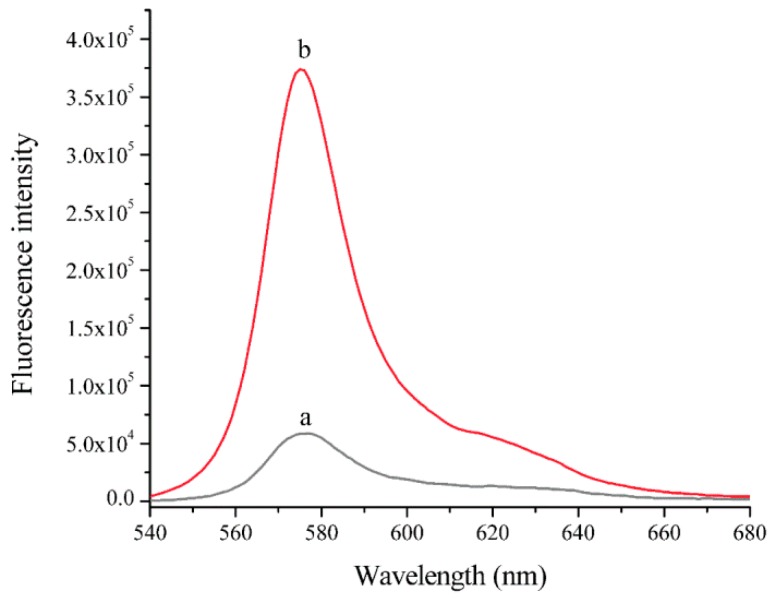
Fluorescence emission spectra of DNAzyme-R-PE incubated with 0 nM (curve a) and 150 nM (curve b) Pb^2+^ in HEPES buffer.

After conjugating the enzyme-substrate complex onto R-PE, we tested the feasibility of DNAzyme-R-PE as a biosensor for detecting Pb^2+^ in aqueous solution. As shown in Figure 2, the DNAzyme-R-PE exhibited minimum fluorescence without Pb^2+^ (curve a), indicating the Iowa Black**^®^** FQ molecules immobilized on R-PE and quenched the fluorescence of R-PE. In contrast, the fluorescence of R-PE was recovered and enhanced by nearly sevenfold in the presence of 150 nM Pb^2+^ (curve b), demonstrating that the enzyme and substrate containing quencher had been cleaved off R-PE. Therefore, the fluorescence change of DNAzyme-R-PE was employed to detect Pb^2+^ in aqueous solution.

### 3.2. Optimization of R-PE-to-complex molar ratio

**Figure 3:**
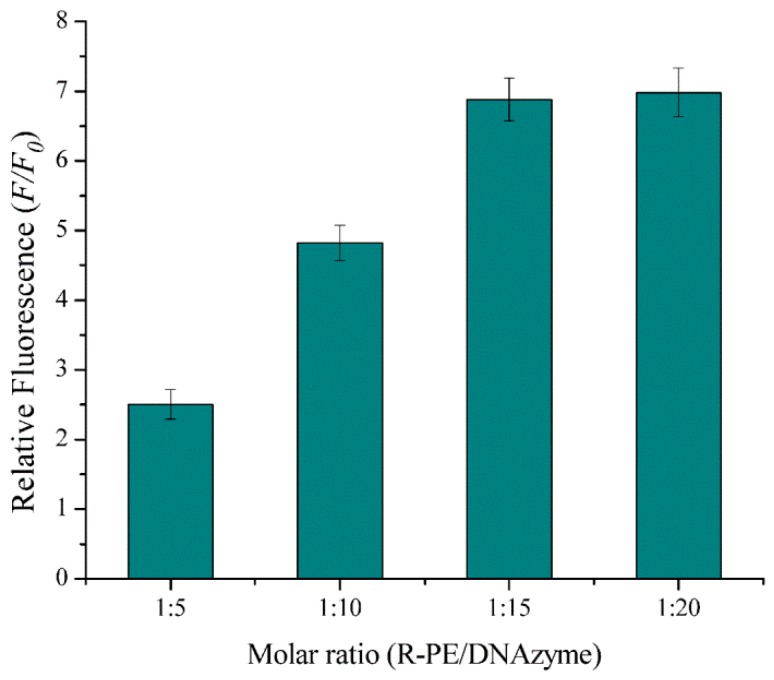
Relative fluorescence intensity of the DNAzymes-R-PE at a different R-PE-to-complex molar ratio. *F*_0_ (*F*) was the fluorescence intensity of DNAzyme-R-PE before (after) the addition of 150 nM Pb^2+^.

R-PE’ surface possesses multiple lysine residues and the maxmum amount of DNAzyme conjugated covalently to R-PE facilitates a reduction in the background, thereby achieving the best performance of the biosensor. Therefore, we optimized the R-PE-to-complex molar ratio for the best performance of the biosensor. The DNAzyme-R-PE was prepared by the reaction of R-PE with 5, 10, 15, and 20 equiv. of the thiol-modified enzyme-substrate complex, respectively. We employed the relative fluorescence intensity (*F*/*F*_0_), where *F*_0_ and *F* represented the fluorescence signal of DNAzyme-R-PE without and with Pb^2+^, to evaluate the performance of DNAzyme-R-PE. As indicated in Figure 3, *F*/*F*_0_ increased with increasing R-PE-to-complex molar ratio from 5 to 20. The maximum *F*/*F*_0_ was obtained in the R-PE-to-complex molar ratio of 1:15. In addition, the molecular ratio of the enzyme-substrate complex versus R-PE in the proposed biosensor was estimated to be ~5 enzyme-substrate complex per R-PE by comparing the absorbance of the biosensor at 260 (DNAzyme-substrate complex) and 565 nm (R-PE), respectively. Thus, in the subsequent experiments, we prepared our biosensor with the R-PE-to-complex molar ratio of 1:15.

### 3.3. Performance of DNAzyme-R-PE

**Figure 4:**
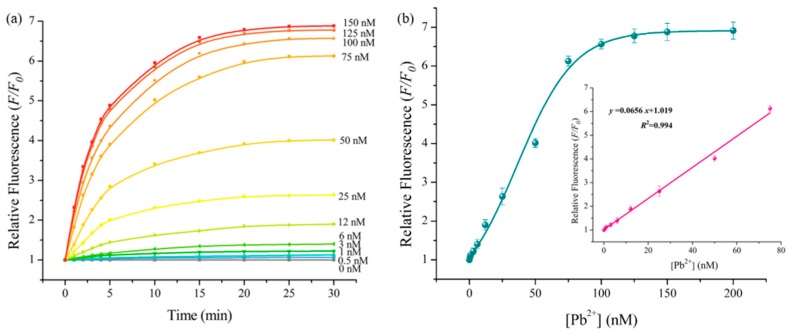
(**a**) Time-dependent fluorescence changes upon analyzing Pb^2+^ (0~150 nM) by DNAzyme-R-PE. (**b**) The relationship between the relative intensity of DNAzyme-R-PE at 20 min and the concentrations of Pb^2+^. Inset: The linear plot of *F*/*F*_0_ vs[Pb^2+^].

We investigated the time-dependent fluorescence changes of DNAzyme-R-PE when it was incubated with various concentrations of Pb^2+^ (Figure 4a). The higher the concentration of Pb^2+^, the greater the rate of increase in relative fluorescence intensity [32], and the relative fluorescence intensity had little change after approximately 20 min. The relative fluorescence intensity at 20 min increased gradually as the concentration of Pb^2+^ increased (Figure 4b). DNAzyme-R-PE exhibited a good linear relationship with the Pb^2+^ concentration in the range of 0.5–75 nM. (Figure 4b, inset). The regression equation was *y* = 1.019 + 0.0656*x* with a correlation coefficient of 0.994, where *y* was *F*/*F*_0_ and *x* was the concentration of Pb^2+^. We estimated the detection limit of DNAzyme-R-PE to be 0.16 nM according to the 3σ rule (3σ/S, σ was the standard deviation of the blank samples (*n* = 11), S referred to the slope of the fitting standard curve). The maximum permissible level for Pb^2+^ in drinking water is 72 nM according to the Enviromental Protection Agency (EPA). Our sensor meets the requirements for routine monitoring Pb^2+^ in water samples. Furthermore, the sensitivity of DNAzyme-R-PE is superior or comparable to assays based on more complex and expensive DNAzymes and different sensing principles (Table S1). For example, Liu et al. improved the sensitivity of Pb^2+^ detection (LOD, 0.1 nM) by incorporating the PS modification to GR-5 DNAzyme, while PS modification increases the DNAzyme cost and reduces the selectivity of GR-5 DNAzyme as compared to our sensor.

### 3.4. The Selectivity of DNAzyme-R-PE

**Figure 5:**
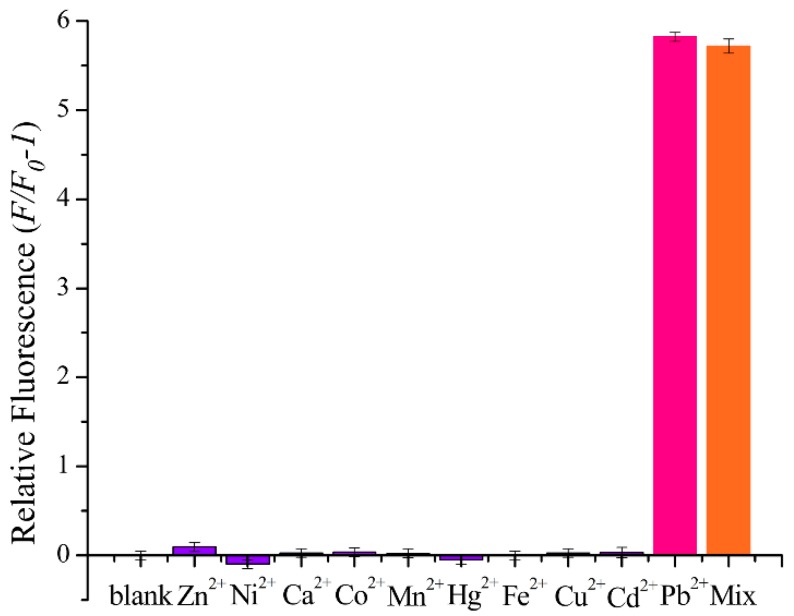
The selectivity of DNAzyme-R-PE for Pb^2+^ (150 nM) over other divalent metal ions (150 nM).

To test the selectivity of the DNAzyme-R-PE for Pb^2+^, DNAzyme-R-PE was challenged with several environmentally divalent metal ions (Ca^2+^, Zn^2+^, Ni^2+^, Co^2+^, Mn^2+^, Hg^2+^, Fe^2+^, Cu^2+^ and Cd^2+^), respectively. As described in Figure 5, only Pb^2+^ could significantly enhance the fluorescence signal of the DNAzyme-R-PE, whereas interfering ions produced a negligible fluorescence signal relative to the blank under the same condition. Moreover, coexistence of other interfering ions does not affect the response of DNAzyme-R-PE to lead ions. These results showed that DNAzyme-R-PE displayed significant selectivity for Pb^2+^ relative to the other divalent metal ions, which was attributed to the specific recognition of GR-5 DNAzyme towards Pb^2+^ [33]. 

### 3.5. Real Sample Detection

The potential application of DNAzyme-R-PE for Pb^2+^ analysis in real samples was evaluated using the standard addition method [34]. The aquatic water samples were collected from mirror lakes in the Shanghai Ocean University campus (Shanghai, China). After centrifugation and filtration, the water samples were spiked with 5.00, 15.00, 45.00 nM Pb^2+^, respectively. These spiked water samples were determined by DNAzyme-R-PE. As shown in Table 1, DNAzyme-R-PE could determine the spiked Pb^2+^ in lake water samples with good recoveries from 98.24% to 102.41%, indicating that the proposed biosensor had good reliability and accuracy for Pb^2+^ detection in complicated samples.

## 4. Conclusions

A novel fluorescent biosensor, DNAzyme-functionalized R-PE, was constructed for Pb^2+^ detection by combining the excellent optical property of R-PE with the high specificity of GR-5 DNAzyme towards Pb^2+^. This sensor can selectively detect Pb^2+^ within 20 min with a low limit of detection of 0.16 nM. Furthermore, we demonstrated that DNAzyme-R-PE could reliably determine Pb^2+^ in spiked lake water. Compared to previously reported DNAzyme-based biosensors, DNAzyme-R-PE has several advantages. First, DNAzyme-R-PE is low-cost because R-PE is efficiently and economically purified from a red alga on a large scale. Second, DNAzyme-R-PE is highly sensitive, originating from the high quantum yield of R-PE and minimal background interference from biological molecules. Third, DNAzyme-R-PE is environmentally friendly. R-PE is non-toxic and the usage of R-PE does not cause secondary pollution to the environment.

## Figures and Tables

**Table 1:** Fluorescent assay of Pb^2+^ in spiked lake water samples using DNAzyme-R-PE (*n* = 5).

Sample	Spiked (nM)	Found (nM, Mean *^a^* ± SD *^b^*)	Recovery (%)
1	5.00	5.11 ± 0.24	102.27%
2	15.00	14.74 ± 0.47	98.24%
3	45.00	46.09 ± 0.44	102.41%

*^a^* Mean: Average value of five individual determinations. *^b^* SD: Standard deviation of five individual determinations.

## Supplementary Materials

The Supplementary Materials are available online at https://www.mdpi.com/1424-8220/19/12/2732/s1, Figure S1: TEM image (negative staining) of R-PE, Figure S2: Native-PAGE (8%) analysis of R-PE and DNAzyme-R-PE, Table S1: Comparison of the performance DNAzyme-based fluorescent biosensors.
