# Supplementary material for: DNAzyme-Functionalized R-Phycoerythrin as a Cost-Effective and Environment-Friendly Fluorescent Biosensor for Aqueous Pb2+ Detection
Source: Sensors (Basel). 2019 Jun 18;19(12):2732. doi: 10.3390/s19122732 (PMC6630308; doi:10.3390/s19122732)
Supplement: Supplementary file 1 [file sensors-19-02732-s001.pdf]

sensors

MDPI

Article

DNAzyme-functionalized R-phycoerythrin as a cost-effective and environment-friendly fluorescent biosensor for aqueous Pb^{2+}detection

Jikui Wu [1,] *, Yunfei Lu [1], Ningna Ren [1], Min Jia [1], Ruinan Wang[1] and Junling Zhang [2,] *

[1] College of Food Science and Technology; Ministry of Agriculture; National R&D Branch Center for Freshwater Aquatic Products Processing Technology (Shanghai); Laboratory of Quality and Safety Risk Assessment for Aquatic Product on Storage and Preservation (Shanghai), Shanghai Ocean University, Shanghai, 201306, China; yifland0929@163.com (Y.L.) ; Ningna_ren@163.com (N.R.); m170200397@st.shou.edu.cn (J.M.); m170200399@st.shou.edu.cn (R.W.)

[2] Laboratory of Freshwater Aquatic Genetic Resources, Ministry of Agriculture; Shanghai Engineering Research Center of Aquaculture; National Demonstration Center for Experimental Fisheries Science Education；Shanghai Ocean University, Shanghai, 201306, China

* Correspondence: jkwu@shou.edu.cn (J.W.); jlzhang@shou.edu.cn (J.Z.); Tel.: +86-021-61900753 (J.W.); Tel.: +86-021-61900473 (J.Z.)

Received: 13 May 2019; Accepted: 16 June 2019; Published: date

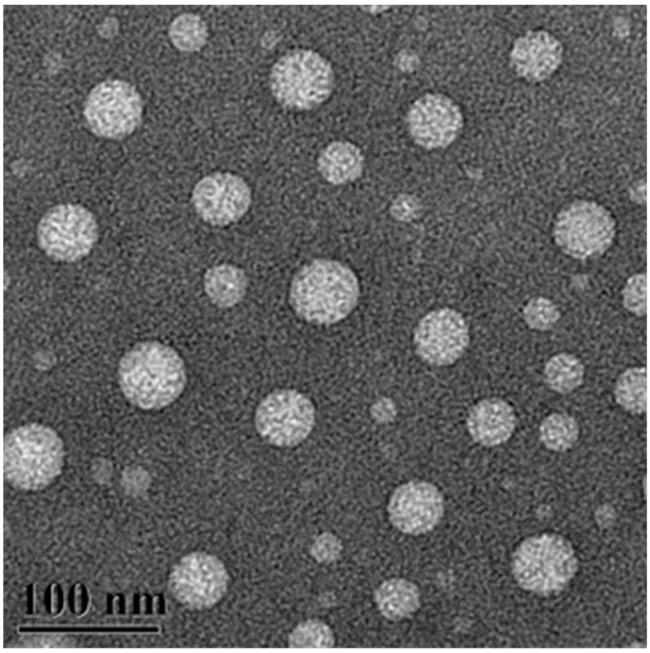

Figure S1. TEM image (negative staining) of R-PE

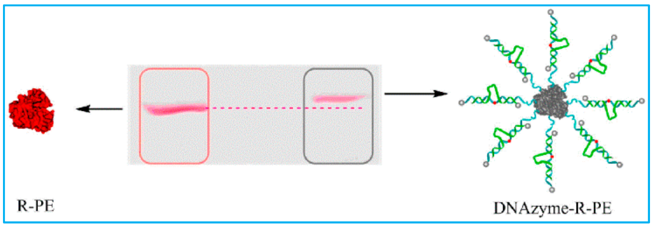

Figure 2. Native-PAGE (8%) analysis of R-PE and DNAzyme-R-PE.

Table 1. Comparison of the performance of biosensors based on different sensing principles for Pb^{2+} detection.

Detection Technique	Recognition Element	Linear Range	LOD	Reference
Colorimetry	GR-5 DNAzyme	1–500 nM	0.7 nM	[1]
Colorimetry	GR-5 DNAzyme	0.1–1000 nM	0.3 nM	[2]
Colorimetry	8-17 DNAzyme	5–100 nM	20 nM	[3]
Electrochemistry	8-17 DNAzyme	0.5–10 μM	0.3 μM	[4]
Electrochemistry	GR-5 DNAzyme	1 nM–10 mM	1 nM	[5]
Electrochemistry	GR-5 DNAzyme	0.05–200 nM	0.034 nM	[6]
Fluorescence	8-17 DNAzyme	10 nM–2.5 mM	1.7 nM	[7]
Fluorescence	GR-5 DNAzyme	1–50 nM	0.2 nM	[8]
Fluorescence	8-17 DNAzyme	5 nM–1 μM	1 nM	[9]
Fluorescence	GR-5 DNAzyme	0.2–2 nM	0.1 nM	[10]
Fluorescence	GR-5 DNAzyme	0.5–75 nM	0.16 nM	This work

References:

1. Zhu, Y.Y.; Deng, D.Q.; Xu, L.G.; Zhu, Y.B.; Wang, L.M.; Qi, B.; Xu, C.L. Ultrasensitive detection of lead ions based on a DNA-labelled DNAzyme sensor. *Anal. Methods* **2015**, *7*, 662–666; doi:10.1039/C4AY02654C.
2. Vijitvarasan, P.; Oaew, S.; Surareungchai, W. Paper-based scanometric assay for lead ion detection using DNAzyme. *Ana. Chim. Acta*. **2015**, *896*, 152–157; doi:10.1016/j.aca.2015.09.011.
3. Wang, Z.; Chen, B.; Duan, J.; Hao, T.; Jiang, X.; Guo, Z.; Wang, S. A test strip for lead (II) based on gold nanoparticles multi-functionalized by DNAzyme and barcode DNA. *J. Am. Chem. Soc.* **2015**, *70*, 339–345; doi:10.1134/S1061934815030247.
4. Yi, X.; Aaron, A.R.; Kevin W. P. Electrochemical detection of parts-per-billion lead via an electrode-bound DNAzyme Assembly. *J. Am. Chem. Soc.* **2007**, *129*, 262–263; doi:10.1021/ja067278x.
5. Fu, C.C.; Xu, W.Q.; Wang, H.L.; Ding, H.; Liang, L.J.; Cong, M.; Xu, S.P. DNAzyme-based plasmonic nanomachine for ultrasensitive selective surface-enhanced Raman scattering detection of lead ions via a particle-on-a-film hot spot construction. *Anal. Chem.* **2014**, *86*, 11494–11497; doi:10.1021/ac5038736.
6. Cui, L.; Wu, J.; Li, J.; Ju, H.X. Electrochemical sensor for lead cation sensitized with a DNA functionalized Porphyrinic metal–organic framework. *Anal. Chem.* **2015**, *87*, 10635–10641; doi:10.1021/acs.analchem.5b03287.
7. Wang, X.Y.; Niu, C.G.; Guo, L.J.; Hu, L.Y.; Wu, S.Q.; Zeng, G.M.; Li, F. A fluorescence sensor for lead (II) ions determination based on label-free gold nanoparticles (GNPs)-DNAzyme using time-gated mode in aqueous solution. *J Fluoresc.* **2017**, *27*, 643–649; doi:10.1007/s10895-016-1993-y.
8. Wu, C.S.; Khaing Oo, M.K.; Fan, X. Highly sensitive multiplexed heavy metal detection using quantum-dot-labeled DNAzymes, *ACS Nano* **2010**, *4*, 5897–5904; doi:10.1021/nn1021988.
9. Yao, J.; Li, J.; Owens, J.; Zhong, W. Combing DNAzyme with single-walled carbon nanotubes for detection of Pb (II) in water, *Analyst* **2011**, *136*, 764–768; doi:10.1039/c0an00709a.
10. Huang, P.J. Liu, J.W. Sensing parts-per-trillion Cd^{2+}, Hg^{2+}, and Pb^{2+} collectively and individually using phosphorothioate DNAzymes. *Anal. Chem.* **2014**, *86*, 5999–6005; doi:10.1021/ac501070a.

© 2019 by the authors. Licensee MDPI, Basel, Switzerland. This article is an open access article distributed under the terms and conditions of the

Creative Commons Attribution (CC BY) license (http://creativecommons.org/licenses/by/4.0/).
